# 2-Iodo-*N*-(6-methyl-2-pyrid­yl)benzamide

**DOI:** 10.1107/S1600536808041950

**Published:** 2008-12-13

**Authors:** Hoong-Kun Fun, Reza Kia, Annada C. Maity, Sibaprasad Maity, Shyamaprosad Goswami

**Affiliations:** a X-ray Crystallography Unit, School of Physics, Universiti Sains Malaysia, 11800 USM, Penang, Malaysia; b Department of Chemistry, Bengal Engineering and Science University, Shibpur, Howrah 711 103, India

## Abstract

The asymmetric unit of the title compound, C_13_H_11_IN_2_O, comprises two crystallographically independent mol­ecules. The dihedral angles between the ring planes are 53.56 (9) and 72.14 (19)° in the two molecules. Pairs of inter­molecular N—H⋯N hydrogen bonds and I⋯O inter­actions link neighbouring mol­ecules into two different pairs of dimers, those involving N—H⋯N interactions having *R* ^2^_2_(8) ring motifs. Short inter­molecular I⋯O [3.1458 (15) Å] and I⋯N [3.4834 (16) Å] contacts are present. The crystal structure is further stabilized by inter­molecular C—H⋯π inter­actions [3.565 (2) and 3.629 (2) Å].

## Related literature

For details of hydrogen-bond motifs, see: Bernstein *et al.* (1995 ▶). For applications in supramolecular chemistry and molecular recognition, see, for example: Goswami & Dey (2006 ▶); Goswami *et al.* (2005**a* ▶,b* ▶); Steed & Atwood (2001 ▶); Lehn (1995 ▶); Desiraju (2003 ▶).
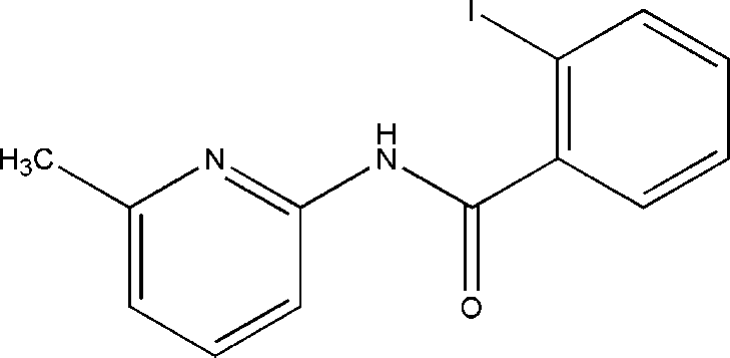


## Experimental

### 

#### Crystal data

C_13_H_11_IN_2_O
*M* *_r_* = 338.14
Triclinic, 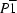

*a* = 9.8687 (3) Å
*b* = 10.1276 (3) Å
*c* = 13.6366 (4) Å
α = 97.521 (1)°
β = 93.113 (1)°
γ = 110.380 (1)°
*V* = 1259.28 (6) Å^3^
*Z* = 4
Mo *K*α radiation
μ = 2.53 mm^−1^
*T* = 100.0 (1) K
0.49 × 0.32 × 0.12 mm

#### Data collection

Bruker SMART APEXII CCD area-detector diffractometer
Absorption correction: multi-scan (**SADABS**; Bruker, 2005 ▶) *T*_min_ = 0.374, *T*_max_ = 0.746
47136 measured reflections
11804 independent reflections
9568 reflections with *I* > 2σ(*I*)
*R*_int_ = 0.036

#### Refinement

*R*[*F* ^2^ > 2σ(*F* ^2^)] = 0.034
*wR*(*F* ^2^) = 0.090
*S* = 1.05
11804 reflections
317 parameters
H atoms treated by a mixture of independent and constrained refinement
Δρ_max_ = 2.34 e Å^−3^
Δρ_min_ = −1.51 e Å^−3^

### 

Data collection: *APEX2* (Bruker, 2005 ▶); cell refinement: *SAINT* (Bruker, 2005 ▶); data reduction: *SAINT*; program(s) used to solve structure: *SHELXTL* (Sheldrick, 2008 ▶); program(s) used to refine structure: *SHELXTL*; molecular graphics: *SHELXTL*; software used to prepare material for publication: *SHELXTL* and *PLATON* (Spek, 2003 ▶).

## Supplementary Material

Crystal structure: contains datablocks global, I. DOI: 10.1107/S1600536808041950/tk2344sup1.cif

Structure factors: contains datablocks I. DOI: 10.1107/S1600536808041950/tk2344Isup2.hkl

Additional supplementary materials:  crystallographic information; 3D view; checkCIF report

## Figures and Tables

**Table 1:** Hydrogen-bond geometry (Å, °)

*D*—H⋯*A*	*D*—H	H⋯*A*	*D*⋯*A*	*D*—H⋯*A*
N1*A*—H1*NA*⋯N2*B*^i^	0.83 (3)	2.14 (3)	2.962 (2)	172 (3)
N1*B*—H1*NB*⋯N2*A*^i^	0.84 (3)	2.25 (3)	3.079 (2)	169 (2)
C9*A*—H9*AA*⋯O1*A*	0.93	2.40	2.895 (2)	113
C5*B*—H5*BA*⋯O1*A*^ii^	0.93	2.58	3.359 (2)	141
C9*B*—H9*BA*⋯O1*B*	0.93	2.26	2.825 (3)	118
C11*A*—H11*A*⋯O1*B*^iii^	0.93	2.48	3.254 (2)	141
C4*B*—H4*BA*⋯*Cg*1^ii^	0.93	2.71	3.565 (2)	153
C13*A*—H13*C*⋯*Cg*2^i^	0.96	2.85	3.629 (2)	139

Symmetry codes: (i) 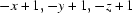; (ii) 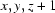; (iii) 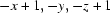. *Cg*1 and *Cg*2 are the centroids of the C1*A*–C6*A* and C1*B*–C6*B* rings, respectively.

**Fig. 1.**
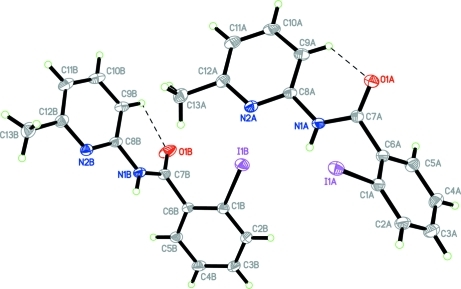
The molecular structure of (I), showing 50% probability displacement ellipsoids and the atomic numbering. Dashed lines show intramolecular hydrogen bonds.

**Fig. 2.**
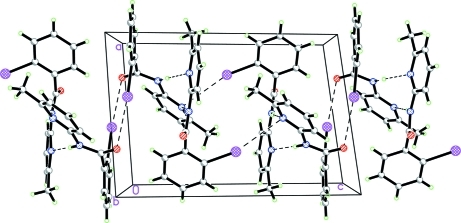
The crystal packing of (I), viewed down the b-axis, showing pairs of dimers with R22(8) motifs linked together through N—H···N interactions and other pairs of dimers linked together by I···O interactions. Intermolecular interactions are drawn as dashed lines.

## supplementary crystallographic information

### Comment

The role of hydrogen bonds involving neutral amidic-type NH and carbonyl type O-atom in the controlled assembly of biologically relevant molecules giving outstanding architectures is an obvious entity in nature. Amide derivatives of heterocyclic compounds are important hydrogen bonding synthons as they are the very useful substrates which can be used for the synthesis of designed receptors in the field of molecular recognition (Goswami & Dey 2006 *a*; Goswami *et al.*, 2005*a*) and supramolecular chemistry (Steed & Atwood 2001; Lehn 1995; Desiraju 2003). We have previously reported the design and synthesis of a series of symmetric diamido biaryls by direct homocoupling of iodoarylbenzamides (Goswmai *et al.* 2005*b*).

In the title compound (I), Fig. 1, intramolecular C—H···O hydrogen bonds generate six-membered rings, producing *S(6)* ring motifs. Pairs of intermolecular N—H···N hydrogen bonds and I···O interactions link neighbouring molecules into two different pairs of dimers, those involving N—H···N interactions having *R^2^_2_(8)* ring motifs (Fig. 2). The interesting features of the crystal structure are the short intermolecular I···O [3.1458 (15) Å; symmetry: 1 - *x*, 1 - *y*, -*z*] and I···N [3.4834 (16) Å; symmetry: *x*, *y*, *z*] contacts which are significanlty shorter than the sum of the van der Waals radii of these atoms. The crystal structure is further stabilized by intermolecular C—H···π interactions (Table 1, *Cg*1 and *Cg*2 are the centroids of the C1A–C6A and C1B–C6B benzene rings).

### Experimental

To a magnetically stirred solution of the 2-amino-6-methylpyridine (108 mg, 1.0 mmol) in dry CH_2_Cl_2_ (20 ml) and freshly distilled triethylamine (1.2 equiv), was added 2-iodobenzoyl chloride (1.1 equiv). Stirring was continued for 12 h. The triethylamine hydrochloride was filtered off, the organic layer after washing with water was dried (anhydrous Na_2_SO_4_) and then the solvent was removed under reduced pressure. The residue was purified using column chromatography on silica-gel to afford (I) (310 mg, 92%).

### Refinement

N-bound hydrogen atoms were located from a difference Fourier map and refined freely; see Table 1 for N—H distances. The remaining H atoms were positioned geometrically with C—H = 0.93 Å (aromatic) or 0.96 Å (methyl), and refined in the riding model approximation with *U*_iso_(H) = 1.2 or 1.5 *U*_eq_(C). A rotating group model was used for the methyl groups. The highest peak (2.34 e. Å^-3^) is located 0.70 Å from I1A and the deepest hole (-1.51 e. Å^-3^) is located 0.75 Å from I1A.

### Crystal data

**Table:**

C_13_H_11_IN_2_O	*Z* = 4
*M**_r_* = 338.14	*F*(000) = 656
Triclinic, *P*1	*D*_x_ = 1.784 Mg m^−^^3^
Hall symbol: -P 1	Mo *K*α radiation, λ = 0.71073 Å
*a* = 9.8687 (3) Å	Cell parameters from 9385 reflections
*b* = 10.1276 (3) Å	θ = 2.5–35.9°
*c* = 13.6366 (4) Å	µ = 2.53 mm^−^^1^
α = 97.521 (1)°	*T* = 100 K
β = 93.113 (1)°	Plate, colourless
γ = 110.380 (1)°	0.49 × 0.32 × 0.12 mm
*V* = 1259.28 (6) Å^3^	

### Data collection

**Table:**

Bruker SMART APEXII CCD area-detector diffractometer	11804 independent reflections
Radiation source: fine-focus sealed tube	9568 reflections with *I* > 2σ(*I*)
graphite	*R*_int_ = 0.036
φ and ω scans	θ_max_ = 36.0°, θ_min_ = 2.2°
Absorption correction: multi-scan (*SADABS*; Bruker, 2005)	*h* = −16→16
*T*_min_ = 0.374, *T*_max_ = 0.746	*k* = −16→16
47136 measured reflections	*l* = −22→21

### Refinement

**Table:**

Refinement on *F*^2^	Primary atom site location: structure-invariant direct methods
Least-squares matrix: full	Secondary atom site location: difference Fourier map
*R*[*F*^2^ > 2σ(*F*^2^)] = 0.034	Hydrogen site location: inferred from neighbouring sites
*wR*(*F*^2^) = 0.090	H atoms treated by a mixture of independent and constrained refinement
*S* = 1.05	*w* = 1/[σ^2^(*F*_o_^2^) + (0.0498*P*)^2^] where *P* = (*F*_o_^2^ + 2*F*_c_^2^)/3
11804 reflections	(Δ/σ)_max_ = 0.002
317 parameters	Δρ_max_ = 2.34 e Å^−^^3^
0 restraints	Δρ_min_ = −1.51 e Å^−^^3^

### Special details

**Table:**

Experimental. The low-temperature data was collected with the Oxford Cyrosystem Cobra low-temperature attachment.
Geometry. All e.s.d.'s (except the e.s.d. in the dihedral angle between two l.s. planes) are estimated using the full covariance matrix. The cell e.s.d.'s are taken into account individually in the estimation of e.s.d.'s in distances, angles and torsion angles; correlations between e.s.d.'s in cell parameters are only used when they are defined by crystal symmetry. An approximate (isotropic) treatment of cell e.s.d.'s is used for estimating e.s.d.'s involving l.s. planes.
Refinement. Refinement of *F*^2^ against ALL reflections. The weighted *R*-factor *wR* and goodness of fit *S* are based on *F*^2^, conventional *R*-factors *R* are based on *F*, with *F* set to zero for negative *F*^2^. The threshold expression of *F*^2^ > σ(*F*^2^) is used only for calculating *R*-factors(gt) *etc*. and is not relevant to the choice of reflections for refinement. *R*-factors based on *F*^2^ are statistically about twice as large as those based on *F*, and *R*- factors based on ALL data will be even larger.

### Fractional atomic coordinates and isotropic or equivalent isotropic displacement parameters (Å^2^)

**Table:**

	*x*	*y*	*z*	*U*_iso_*/*U*_eq_	
I1A	0.600768 (13)	0.719329 (12)	0.055937 (8)	0.02055 (4)	
O1A	0.73426 (15)	0.40166 (14)	0.01538 (10)	0.0225 (3)	
N1A	0.71156 (17)	0.46318 (15)	0.17981 (11)	0.0178 (3)	
N2A	0.53614 (17)	0.34154 (15)	0.27329 (11)	0.0179 (3)	
C1A	0.8221 (2)	0.75540 (18)	0.08834 (12)	0.0181 (3)	
C2A	0.9243 (2)	0.89336 (19)	0.09553 (14)	0.0229 (3)	
H2AA	0.8937	0.9700	0.0928	0.027*	
C3A	1.0713 (2)	0.9156 (2)	0.10670 (14)	0.0246 (4)	
H3AA	1.1393	1.0077	0.1128	0.030*	
C4A	1.1178 (2)	0.8017 (2)	0.10882 (14)	0.0245 (4)	
H4AA	1.2166	0.8169	0.1141	0.029*	
C5A	1.0165 (2)	0.6648 (2)	0.10306 (14)	0.0228 (3)	
H5AA	1.0479	0.5885	0.1049	0.027*	
C6A	0.86799 (19)	0.64057 (17)	0.09455 (12)	0.0176 (3)	
C7A	0.76383 (19)	0.49040 (17)	0.09119 (13)	0.0174 (3)	
C8A	0.61757 (19)	0.32996 (17)	0.19928 (12)	0.0170 (3)	
C9A	0.6157 (2)	0.20087 (18)	0.14923 (14)	0.0218 (3)	
H9AA	0.6738	0.1973	0.0983	0.026*	
C10A	0.5238 (2)	0.07761 (19)	0.17815 (16)	0.0260 (4)	
H10A	0.5191	−0.0112	0.1464	0.031*	
C11A	0.4392 (2)	0.08663 (19)	0.25418 (15)	0.0243 (4)	
H11A	0.3774	0.0043	0.2741	0.029*	
C12A	0.4472 (2)	0.22025 (19)	0.30064 (14)	0.0206 (3)	
C13A	0.3562 (2)	0.2346 (2)	0.38233 (16)	0.0267 (4)	
H13A	0.4006	0.3270	0.4226	0.040*	
H13B	0.3485	0.1620	0.4227	0.040*	
H13C	0.2610	0.2241	0.3543	0.040*	
I1B	0.755985 (14)	0.434720 (13)	0.498669 (9)	0.02504 (4)	
O1B	0.64755 (17)	0.24584 (15)	0.71745 (14)	0.0330 (4)	
N1B	0.49390 (17)	0.36405 (15)	0.68719 (11)	0.0176 (3)	
N2B	0.24463 (17)	0.28564 (15)	0.66686 (11)	0.0182 (3)	
C1B	0.8325 (2)	0.53935 (18)	0.64543 (13)	0.0194 (3)	
C2B	0.9594 (2)	0.65908 (19)	0.66103 (14)	0.0221 (3)	
H2BA	1.0122	0.6876	0.6082	0.027*	
C3B	1.0066 (2)	0.7357 (2)	0.75644 (14)	0.0229 (3)	
H3BA	1.0923	0.8151	0.7677	0.027*	
C4B	0.9267 (2)	0.6946 (2)	0.83480 (14)	0.0244 (4)	
H4BA	0.9578	0.7474	0.8983	0.029*	
C5B	0.7997 (2)	0.57396 (19)	0.81873 (14)	0.0217 (3)	
H5BA	0.7461	0.5464	0.8714	0.026*	
C6B	0.75314 (19)	0.49482 (17)	0.72370 (12)	0.0170 (3)	
C7B	0.6259 (2)	0.35554 (18)	0.70882 (13)	0.0199 (3)	
C8B	0.3617 (2)	0.24816 (17)	0.66336 (12)	0.0180 (3)	
C9B	0.3545 (2)	0.10825 (18)	0.63597 (14)	0.0222 (3)	
H9BA	0.4381	0.0859	0.6336	0.027*	
C10B	0.2170 (2)	0.0036 (2)	0.61241 (15)	0.0265 (4)	
H10B	0.2071	−0.0914	0.5948	0.032*	
C11B	0.0950 (2)	0.0406 (2)	0.61509 (14)	0.0257 (4)	
H11B	0.0027	−0.0291	0.5998	0.031*	
C12B	0.1116 (2)	0.18363 (19)	0.64094 (13)	0.0214 (3)	
C13B	−0.0153 (2)	0.2316 (2)	0.63969 (16)	0.0282 (4)	
H13D	0.0075	0.3177	0.6866	0.042*	
H13E	−0.0983	0.1587	0.6574	0.042*	
H13F	−0.0367	0.2494	0.5742	0.042*	
H1NA	0.728 (3)	0.530 (3)	0.2269 (19)	0.030 (7)*	
H1NB	0.485 (3)	0.444 (3)	0.6892 (19)	0.035 (7)*	

### Atomic displacement parameters (Å^2^)

**Table:**

	*U*^11^	*U*^22^	*U*^33^	*U*^12^	*U*^13^	*U*^23^
I1A	0.02262 (6)	0.01832 (5)	0.02205 (6)	0.00989 (4)	0.00152 (4)	0.00112 (4)
O1A	0.0268 (7)	0.0187 (6)	0.0190 (6)	0.0057 (5)	0.0032 (5)	−0.0012 (5)
N1A	0.0217 (7)	0.0118 (5)	0.0179 (6)	0.0038 (5)	0.0027 (5)	0.0008 (5)
N2A	0.0194 (7)	0.0138 (6)	0.0189 (6)	0.0039 (5)	0.0009 (5)	0.0029 (5)
C1A	0.0212 (8)	0.0158 (7)	0.0170 (7)	0.0062 (6)	0.0022 (6)	0.0022 (5)
C2A	0.0295 (10)	0.0152 (7)	0.0217 (8)	0.0057 (7)	0.0019 (7)	0.0021 (6)
C3A	0.0274 (10)	0.0179 (7)	0.0231 (8)	0.0018 (7)	−0.0004 (7)	0.0034 (6)
C4A	0.0205 (9)	0.0229 (8)	0.0267 (9)	0.0033 (7)	0.0022 (7)	0.0047 (7)
C5A	0.0220 (9)	0.0209 (8)	0.0259 (8)	0.0076 (7)	0.0028 (7)	0.0051 (7)
C6A	0.0202 (8)	0.0150 (6)	0.0167 (7)	0.0055 (6)	0.0019 (6)	0.0018 (5)
C7A	0.0187 (8)	0.0151 (6)	0.0189 (7)	0.0067 (6)	0.0026 (6)	0.0027 (5)
C8A	0.0198 (8)	0.0126 (6)	0.0180 (7)	0.0055 (6)	−0.0002 (6)	0.0023 (5)
C9A	0.0269 (9)	0.0141 (7)	0.0255 (8)	0.0093 (6)	0.0020 (7)	0.0013 (6)
C10A	0.0304 (10)	0.0119 (7)	0.0344 (10)	0.0075 (7)	0.0004 (8)	0.0004 (7)
C11A	0.0249 (9)	0.0140 (7)	0.0322 (10)	0.0045 (6)	−0.0003 (7)	0.0054 (7)
C12A	0.0189 (8)	0.0165 (7)	0.0260 (8)	0.0051 (6)	0.0007 (6)	0.0065 (6)
C13A	0.0278 (10)	0.0204 (8)	0.0317 (10)	0.0062 (7)	0.0077 (8)	0.0085 (7)
I1B	0.02984 (7)	0.02357 (6)	0.01716 (6)	0.00598 (5)	0.00087 (4)	−0.00172 (4)
O1B	0.0255 (7)	0.0141 (6)	0.0585 (10)	0.0062 (5)	−0.0030 (7)	0.0086 (6)
N1B	0.0201 (7)	0.0113 (5)	0.0198 (6)	0.0037 (5)	0.0015 (5)	0.0023 (5)
N2B	0.0196 (7)	0.0167 (6)	0.0161 (6)	0.0040 (5)	0.0008 (5)	0.0026 (5)
C1B	0.0211 (8)	0.0171 (7)	0.0183 (7)	0.0057 (6)	0.0016 (6)	0.0010 (6)
C2B	0.0214 (8)	0.0192 (7)	0.0219 (8)	0.0029 (6)	0.0045 (6)	0.0016 (6)
C3B	0.0209 (9)	0.0184 (7)	0.0237 (8)	0.0017 (6)	0.0000 (6)	0.0000 (6)
C4B	0.0308 (10)	0.0172 (7)	0.0187 (8)	0.0024 (7)	−0.0008 (7)	−0.0004 (6)
C5B	0.0279 (9)	0.0169 (7)	0.0184 (7)	0.0054 (7)	0.0037 (6)	0.0032 (6)
C6B	0.0177 (8)	0.0129 (6)	0.0192 (7)	0.0040 (6)	0.0009 (6)	0.0026 (5)
C7B	0.0221 (8)	0.0142 (7)	0.0226 (8)	0.0053 (6)	0.0030 (6)	0.0034 (6)
C8B	0.0231 (8)	0.0128 (6)	0.0155 (7)	0.0031 (6)	0.0006 (6)	0.0028 (5)
C9B	0.0246 (9)	0.0132 (7)	0.0253 (8)	0.0037 (6)	−0.0006 (7)	0.0005 (6)
C10B	0.0320 (11)	0.0139 (7)	0.0265 (9)	0.0014 (7)	−0.0041 (8)	0.0014 (6)
C11B	0.0241 (9)	0.0190 (8)	0.0252 (9)	−0.0023 (7)	−0.0029 (7)	0.0036 (7)
C12B	0.0219 (9)	0.0206 (8)	0.0174 (7)	0.0023 (6)	−0.0002 (6)	0.0038 (6)
C13B	0.0203 (9)	0.0316 (10)	0.0284 (9)	0.0053 (8)	−0.0006 (7)	0.0023 (8)

### Geometric parameters (Å, °)

**Table:**

I1A—C1A	2.0966 (18)	I1B—C1B	2.1027 (17)
O1A—C7A	1.226 (2)	O1B—C7B	1.221 (2)
N1A—C7A	1.363 (2)	N1B—C7B	1.354 (2)
N1A—C8A	1.414 (2)	N1B—C8B	1.404 (2)
N1A—H1NA	0.83 (3)	N1B—H1NB	0.84 (3)
N2A—C8A	1.342 (2)	N2B—C8B	1.337 (2)
N2A—C12A	1.350 (2)	N2B—C12B	1.351 (2)
C1A—C2A	1.397 (2)	C1B—C6B	1.388 (2)
C1A—C6A	1.398 (2)	C1B—C2B	1.389 (3)
C2A—C3A	1.386 (3)	C2B—C3B	1.389 (3)
C2A—H2AA	0.9300	C2B—H2BA	0.9300
C3A—C4A	1.385 (3)	C3B—C4B	1.384 (3)
C3A—H3AA	0.9300	C3B—H3BA	0.9300
C4A—C5A	1.388 (3)	C4B—C5B	1.395 (3)
C4A—H4AA	0.9300	C4B—H4BA	0.9300
C5A—C6A	1.394 (3)	C5B—C6B	1.393 (2)
C5A—H5AA	0.9300	C5B—H5BA	0.9300
C6A—C7A	1.503 (2)	C6B—C7B	1.507 (2)
C8A—C9A	1.387 (2)	C8B—C9B	1.393 (2)
C9A—C10A	1.384 (3)	C9B—C10B	1.389 (3)
C9A—H9AA	0.9300	C9B—H9BA	0.9300
C10A—C11A	1.379 (3)	C10B—C11B	1.381 (3)
C10A—H10A	0.9300	C10B—H10B	0.9300
C11A—C12A	1.391 (3)	C11B—C12B	1.394 (3)
C11A—H11A	0.9300	C11B—H11B	0.9300
C12A—C13A	1.492 (3)	C12B—C13B	1.493 (3)
C13A—H13A	0.9600	C13B—H13D	0.9600
C13A—H13B	0.9600	C13B—H13E	0.9600
C13A—H13C	0.9600	C13B—H13F	0.9600
			
C7A—N1A—C8A	125.92 (15)	C7B—N1B—C8B	125.89 (15)
C7A—N1A—H1NA	120.2 (18)	C7B—N1B—H1NB	120.7 (19)
C8A—N1A—H1NA	113.6 (18)	C8B—N1B—H1NB	113.4 (19)
C8A—N2A—C12A	117.99 (15)	C8B—N2B—C12B	118.81 (16)
C2A—C1A—C6A	120.10 (17)	C6B—C1B—C2B	121.01 (16)
C2A—C1A—I1A	119.55 (13)	C6B—C1B—I1B	120.61 (13)
C6A—C1A—I1A	120.14 (13)	C2B—C1B—I1B	118.26 (13)
C3A—C2A—C1A	119.77 (17)	C3B—C2B—C1B	119.29 (17)
C3A—C2A—H2AA	120.1	C3B—C2B—H2BA	120.4
C1A—C2A—H2AA	120.1	C1B—C2B—H2BA	120.4
C4A—C3A—C2A	120.51 (18)	C4B—C3B—C2B	120.32 (17)
C4A—C3A—H3AA	119.7	C4B—C3B—H3BA	119.8
C2A—C3A—H3AA	119.7	C2B—C3B—H3BA	119.8
C3A—C4A—C5A	119.77 (18)	C3B—C4B—C5B	120.11 (17)
C3A—C4A—H4AA	120.1	C3B—C4B—H4BA	119.9
C5A—C4A—H4AA	120.1	C5B—C4B—H4BA	119.9
C4A—C5A—C6A	120.64 (17)	C6B—C5B—C4B	119.94 (17)
C4A—C5A—H5AA	119.7	C6B—C5B—H5BA	120.0
C6A—C5A—H5AA	119.7	C4B—C5B—H5BA	120.0
C5A—C6A—C1A	119.12 (16)	C1B—C6B—C5B	119.29 (16)
C5A—C6A—C7A	118.07 (15)	C1B—C6B—C7B	120.77 (15)
C1A—C6A—C7A	122.80 (16)	C5B—C6B—C7B	119.71 (15)
O1A—C7A—N1A	124.45 (16)	O1B—C7B—N1B	125.15 (17)
O1A—C7A—C6A	121.70 (16)	O1B—C7B—C6B	119.13 (17)
N1A—C7A—C6A	113.81 (14)	N1B—C7B—C6B	115.71 (14)
N2A—C8A—C9A	123.90 (16)	N2B—C8B—C9B	123.56 (16)
N2A—C8A—N1A	113.34 (14)	N2B—C8B—N1B	113.63 (14)
C9A—C8A—N1A	122.70 (17)	C9B—C8B—N1B	122.78 (17)
C10A—C9A—C8A	117.33 (18)	C10B—C9B—C8B	117.12 (18)
C10A—C9A—H9AA	121.3	C10B—C9B—H9BA	121.4
C8A—C9A—H9AA	121.3	C8B—C9B—H9BA	121.4
C11A—C10A—C9A	119.90 (17)	C11B—C10B—C9B	120.02 (18)
C11A—C10A—H10A	120.1	C11B—C10B—H10B	120.0
C9A—C10A—H10A	120.1	C9B—C10B—H10B	120.0
C10A—C11A—C12A	119.28 (18)	C10B—C11B—C12B	119.32 (17)
C10A—C11A—H11A	120.4	C10B—C11B—H11B	120.3
C12A—C11A—H11A	120.4	C12B—C11B—H11B	120.3
N2A—C12A—C11A	121.60 (18)	N2B—C12B—C11B	121.10 (18)
N2A—C12A—C13A	117.44 (16)	N2B—C12B—C13B	116.96 (17)
C11A—C12A—C13A	120.95 (17)	C11B—C12B—C13B	121.93 (17)
C12A—C13A—H13A	109.5	C12B—C13B—H13D	109.5
C12A—C13A—H13B	109.5	C12B—C13B—H13E	109.5
H13A—C13A—H13B	109.5	H13D—C13B—H13E	109.5
C12A—C13A—H13C	109.5	C12B—C13B—H13F	109.5
H13A—C13A—H13C	109.5	H13D—C13B—H13F	109.5
H13B—C13A—H13C	109.5	H13E—C13B—H13F	109.5
			
C6A—C1A—C2A—C3A	1.4 (3)	C6B—C1B—C2B—C3B	−0.5 (3)
I1A—C1A—C2A—C3A	−173.36 (14)	I1B—C1B—C2B—C3B	175.59 (14)
C1A—C2A—C3A—C4A	1.3 (3)	C1B—C2B—C3B—C4B	−1.1 (3)
C2A—C3A—C4A—C5A	−2.2 (3)	C2B—C3B—C4B—C5B	1.3 (3)
C3A—C4A—C5A—C6A	0.4 (3)	C3B—C4B—C5B—C6B	0.0 (3)
C4A—C5A—C6A—C1A	2.2 (3)	C2B—C1B—C6B—C5B	1.8 (3)
C4A—C5A—C6A—C7A	−178.51 (17)	I1B—C1B—C6B—C5B	−174.20 (13)
C2A—C1A—C6A—C5A	−3.1 (3)	C2B—C1B—C6B—C7B	−172.64 (17)
I1A—C1A—C6A—C5A	171.60 (13)	I1B—C1B—C6B—C7B	11.3 (2)
C2A—C1A—C6A—C7A	177.67 (16)	C4B—C5B—C6B—C1B	−1.5 (3)
I1A—C1A—C6A—C7A	−7.6 (2)	C4B—C5B—C6B—C7B	172.96 (17)
C8A—N1A—C7A—O1A	0.8 (3)	C8B—N1B—C7B—O1B	−5.4 (3)
C8A—N1A—C7A—C6A	−176.93 (15)	C8B—N1B—C7B—C6B	175.15 (16)
C5A—C6A—C7A—O1A	−77.9 (2)	C1B—C6B—C7B—O1B	86.5 (2)
C1A—C6A—C7A—O1A	101.3 (2)	C5B—C6B—C7B—O1B	−87.9 (2)
C5A—C6A—C7A—N1A	99.94 (19)	C1B—C6B—C7B—N1B	−94.0 (2)
C1A—C6A—C7A—N1A	−80.8 (2)	C5B—C6B—C7B—N1B	91.6 (2)
C12A—N2A—C8A—C9A	0.1 (3)	C12B—N2B—C8B—C9B	−1.2 (3)
C12A—N2A—C8A—N1A	−177.17 (15)	C12B—N2B—C8B—N1B	177.04 (14)
C7A—N1A—C8A—N2A	−153.70 (16)	C7B—N1B—C8B—N2B	165.94 (16)
C7A—N1A—C8A—C9A	29.0 (3)	C7B—N1B—C8B—C9B	−15.8 (3)
N2A—C8A—C9A—C10A	0.0 (3)	N2B—C8B—C9B—C10B	−0.7 (3)
N1A—C8A—C9A—C10A	177.00 (17)	N1B—C8B—C9B—C10B	−178.83 (17)
C8A—C9A—C10A—C11A	−0.2 (3)	C8B—C9B—C10B—C11B	1.1 (3)
C9A—C10A—C11A—C12A	0.2 (3)	C9B—C10B—C11B—C12B	0.4 (3)
C8A—N2A—C12A—C11A	0.0 (3)	C8B—N2B—C12B—C11B	2.8 (3)
C8A—N2A—C12A—C13A	−179.40 (16)	C8B—N2B—C12B—C13B	−176.19 (16)
C10A—C11A—C12A—N2A	−0.1 (3)	C10B—C11B—C12B—N2B	−2.4 (3)
C10A—C11A—C12A—C13A	179.25 (17)	C10B—C11B—C12B—C13B	176.50 (18)

### Hydrogen-bond geometry (Å, °)

**Table:**

*D*—H···*A*	*D*—H	H···*A*	*D*···*A*	*D*—H···*A*
N1A—H1NA···N2B^i^	0.83 (3)	2.14 (3)	2.962 (2)	172 (3)
N1B—H1NB···N2A^i^	0.84 (3)	2.25 (3)	3.079 (2)	169 (2)
C9A—H9AA···O1A	0.93	2.40	2.895 (2)	113
C5B—H5BA···O1A^ii^	0.93	2.58	3.359 (2)	141
C9B—H9BA···O1B	0.93	2.26	2.825 (3)	118
C11A—H11A···O1B^iii^	0.93	2.48	3.254 (2)	141
C4B—H4BA···Cg1^ii^	0.93	2.71	3.565 (2)	153
C13A—H13C···Cg2^i^	0.96	2.85	3.629 (2)	139

Symmetry codes: (i) −*x*+1, −*y*+1, −*z*+1; (ii) *x*, *y*, *z*+1; (iii) −*x*+1, −*y*, −*z*+1.
